# Microvascular Changes in the Retina Correlate with MRI Markers in Patients with Early-Onset Dementia

**DOI:** 10.3390/brainsci12101391

**Published:** 2022-10-14

**Authors:** Ziyi Zhang, Peng Liu, William Robert Kwapong, Bo Wu, Ming Liu, Shuting Zhang

**Affiliations:** 1 Department of Neurology, West China Hospital, Sichuan University, No. 37 Guo Xue Xiang, Chengdu 610041, China; 2 Department of Emergency, West China Hospital, Sichuan University, No. 37 Guo Xue Xiang, Chengdu 610041, China

**Keywords:** retinal microvasculature, optical coherence tomography angiography, early-onset dementia, white matter hyperintensity, medial temporal lobe atrophy

## Abstract

**Background and Aims:** Recent reports suggest that results from imaging retinal microvascular changes with optical coherence tomography angiography (OCTA) in dementia patients reflect cerebral microcirculation changes that occur during dementia. Macula microvascular impairment has been shown in dementia patients compared to controls, but very little is known about its correlation with radiological visual rating scores associated with dementia. We aimed to explore the association between retinal microvasculature and radiological visual rating in early-onset dementia (EOD) patients. **Methods:** Swept-source OCTA (SS-OCTA) was used to image the retinal microvasculature of all EOD patients. Automated software in the OCTA tool segmented and measured the densities in the superficial vascular plexus (SVC) and deep vascular plexus (DVC) and foveal avascular zone (FAZ) areas. Radiological visual rating scores were evaluated on all MR images. **Results:** Medial temporal lobe atrophy (MTA) scores significantly correlated with FAZ area (*p* = 0.031) in EOD patients after adjusting for risk factors. PWMH correlated with SVC (*p* = 0.032) while DWMH significantly correlated with SVC (*p* = 0.007), DVC (*p* = 0.018) and FAZ (*p* = 0.001) in EOD patients. **Discussion:** FAZ changes correlated with MTA scores in EOD patients, while retinal microvasculature correlated with white matter hyperintensity. Our report suggests that microvascular changes in the retina may reflect cortical changes in the brain of EOD patients.

## 1. Introduction

There is currently no treatment for dementia. Clinical trials with drug therapies have been unsuccessful in showing valuable clinical results. However, these studies were done with participants in the advanced phase of the disease when these drugs may not have been effective in slowing down the progression of the disease. Therefore, researchers and clinicians are focused on investigating the earliest phase of dementia to understand the progression of the disease. Early-onset dementia (EOD) is defined as dementia in individuals aged 65 years or younger, and has been suggested to be a model for understanding disease progression and pathophysiology in dementia. 

Structural imaging based on magnetic resonance imaging is an integral part of the clinical assessment of patients with dementia [1]. Many reports [1,2,3] suggest that significant changes occur in cerebral structural markers from the preclinical phase to the overt phase of dementia. Atrophy of medial temporal structures is now considered to be a valid diagnostic marker of dementia. Structural imaging is also included in diagnostic criteria for dementia [4,5]. Cerebral imaging markers such as the presence of atrophy and white matter hyperintensities are sensitive markers of neurodegeneration in dementia [6,7] and are increasingly used as outcome measures in trials of potentially disease-modifying therapies. Recent reports [8,9] suggest that these cerebral imaging markers occur as a result of cerebral microvascular impairment. 

Microvascular impairment is a key pathological feature of dementia, and is likely to be the main pathological driver of disease progression [10,11]. Detecting and monitoring microvascular dysfunction from the earliest phase of dementia is important for identifying patients at risk of dementia, with brain atrophy the current radiological indicator. Brain atrophy is suggested to result from neurodegeneration and can be assessed by visual rating of magnetic resonance imaging (MRI); however, recent reports [10,12] suggest that microvascular dysfunction plays a vital role in brain atrophy. Because of the inconspicuous nature of cerebral microcirculation, its visualization is limited. Furthermore, MRI visual ratings lack sensitivity and do not provide specific information regarding brain regions that are exhibiting accelerated microvascular dysfunction.

Optical coherence tomography angiography (OCTA) is a retinal imaging modality that can non-invasively measure the microvasculature of the retina in different layers with high resolution. The application of OCTA to neurological disorders has been widely documented and has been suggested to reflect cerebral microvascular dysfunction and a surrogate indicator of microvascular impairment in most neurological disorders [13]. OCTA studies have shown decreased microvascular density, reduced fractal dimension, and decreased perfusion in dementia compared to controls [14,15,16,17]; importantly, studies [18,19,20,21] have shown microvascular changes during mild cognitive impairment (MCI) and dementia compared with controls. Although retinal structural changes have been suggested to be linked with cerebral atrophy in dementia, very little is known about the association between radiological indicators of dementia and microvasculature.

In this study, we examined the correlation between retinal microvasculature in early-onset dementia (EOD) and radiological markers.

## 2. Methods

### 2.1. Participants and Study Design

This observational cross-sectional study design was done between September 2021 and July 2022 at the Neurology Department of the West China Hospital, Sichuan University, China. We recruited patients from our ongoing study on Dementia and Aging Project. Patients diagnosed with early-onset dementia (EOD) who fulfilled the NIA-AA criteria [22] and EOD diagnostic criteria [23] were enrolled in our study. Participants enrolled were able to cooperate and tolerate MR imaging and OCTA imaging. Exclusion criteria were as follows: history of other neurological or major psychiatric disorder, presence of cerebral infarction/infarcts on MR imaging, a toxic disorder that can affect the central nervous system (CNS), and aphasia. 

The study was approved by the Ethics Committee of West China Hospital (Ethics approval number: 2020-104) and followed the Declaration of Helsinki. Written informed consent was obtained from each patient before enrollment.

### 2.2. MRI Protocols and Imaging Analysis

MRI was performed on a 3-T MR system (Magnetom Trio, Siemens Medical Systems, Erlangen, Germany). A standardized protocol was used in all patients including Tl-weighted images, T2-weighted images, fluid-attenuated inversion recovery (FLAIR) images, DWI, three-dimensional time-of-flight MRA (3D-TOF-MRA), and susceptibility-weighted image (SWI), as previously reported [24].

Periventricular white matter hyperintensities (PWMH) and deep white matter hyperintensities (DWMH) according to the Standards for Reporting Vascular changes on neuroimaging (STRIVE) consensus criteria [25]; both PWMH and DWMH were evaluated according to the Fazekas scale using FLAIR images [26]. WMH was defined as a high signal intensity region on the FLAIR sequence. The extent of periventricular and deep WMH was rated using the Fazekas scale, where periventricular WMH extends into the deep white matter (Fazekas score 3), or deep WMH (Fazekas score 2 or 3) was regarded as severe WMH. Medial temporal lobe atrophy (MTA), parietal cortical atrophy (PCA), and global cortical atrophy (GCA) were visually rated on MRI scans, as previously reported [27,28]. Cerebral amyloid angiopathy was defined as regions of low-signal blooming artifact on T2 sequences, as previously reported [29].

### 2.3. Swept-Source Optical Coherence Tomography Angiography Imaging

**Figure 1:**
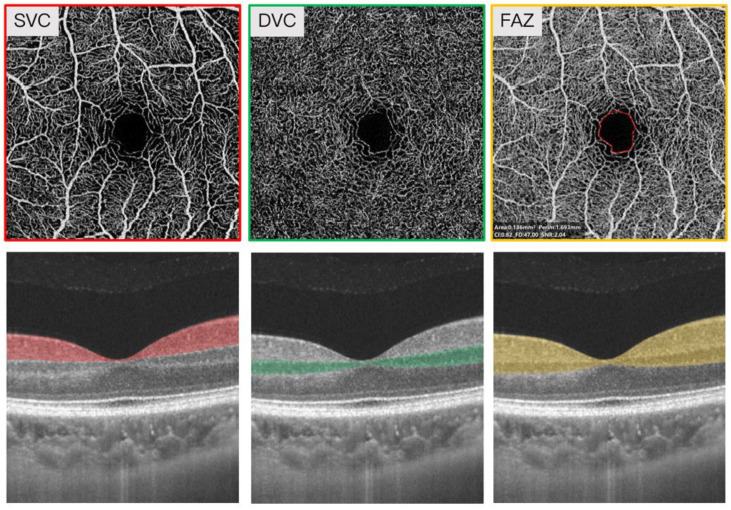
Representative 3 × 3 mm image of the superficial vascular complex (SVC), deep vascular complex (DVC), and fovea avascular zone (FAZ) area around the fovea. The angiograms of the SVC, DVC, and FAZ area were automatically generated by the OCTA tool. The segmentation of the SVC and DVC was set in the inner two-thirds and outer one-third border of the ganglion cell inner plexiform layer (GCIPL). The top panel represents the en face angiograms of SVC, DVC, FAZ while the lower panel represents the segmentation of SVC, DVC, FAZ.

Our previous report described the specification of the SS-OCTA tool [30]. The OCTA images covered an area of 3 × 3 mm^2^ centered on the fovea. The en face angiograms of the superficial vascular complex (SVC) and deep vascular complex (DVC) were generated by the OCTA tool. The segmentation of the SVC and DVC was set in the inner two-thirds and outer one-third border of GCIPL as shown in Figure 1. Mean percentages (%) of the microvasculature in the SVC and DVC were obtained with an in-built algorithm in the OCTA tool. The fovea avascular zone (FAZ) area was automatically generated by the OCTA tool. Angiograms with poor imaging quality (signal quality less than 7), artifacts, presence of severe cataract (which could be detected by the OCTA tool), microcystic macular edema (MME), and age-macular degeneration, were excluded.

### 2.4. Statistical analyses

SPSS Statistics (version 24, IBM, NY, USA) was used to perform all statistical analyses. The Shapiro-Wilk test was used to assess normality of the data. Continuous variables with normal distributions were expressed as mean ± standard deviations while skewed distributions were expressed as medians and interquartile ranges. A multiple linear regression model with generalized linear equations (GEE) was performed to assess the association between SS-OCTA parameters and MRI visual scores while adjusting for risk factors (age, gender, hypertension, diabetes, and CAA). A *p*-value less than 0.05 (*p* < 0.05) was considered statistically significant. 

## 3. Results

Eighty-three EOD patients were initially enrolled in our study; however, seven could not complete MR imaging while thirteen could not meet our SS-OCTA inclusion criteria (five had severe cataracts, six had age-related macular degeneration and two had cataract surgery 2 months ago). 

Our data analysis included 63 EOD patients (mean age: 60.43 ± 5.80 years; 42.86 % males); 11 (17.46 %) had hypertension while 4 (6.34 %) had diabetes. Table 1 shows the clinical information, SS-OCTA results, and radiological information of the EOD patients. 

Table 2 shows the correlation between SS-OCTA parameters and radiological markers in EOD patients. We showed FAZ area significantly correlated with (*p* = 0.031) MTA scores in EOD patients. PWMH correlated with SVC (*p* = 0.032) while DWMH significantly correlated with SVC (*p* = 0.007), DVC (*p* = 0.018) and FAZ (*p* = 0.001) in EOD patients.

## 4. Discussion

Several reports [14,15,16] have shown retinal microvascular changes using OCTA in preclinical AD and AD dementia, some reports have shown significant microvascular changes in AD dementia compared to controls, and some reports did not find any significant differences in the retinal microvasculature. The inconsistencies in previous studies may be due to diagnostic criteria that differed between studies. In addition, most of these studies were limited by small sample sizes. Using a swept-source OCTA (SS-OCTA), we showed that EOD patients had significantly reduced microvascular densities and enlarged FAZ area compared to controls. We suggest that the microvascular changes seen in our study may reflect the cerebral microcirculation changes in EOD. 

A novel finding was the significant correlation between MTA and FAZ area in EOD patients. Recent reports found a large proportion of patients diagnosed with dementia have a combination of neurodegeneration and vascular pathology in the brain [10,31]. From a radiological standpoint, MTA is usually thought to be a substitute marker of neurodegeneration because it occurs in the medial temporal lobe [32]. On the other hand, MTA may be due to vascular pathology, and more specifically to small vessel disease and ischemia, which have been reported to be implicated in the development of dementia [10,33]. Similarly, OCTA reports have shown enlarged FAZ area in dementia patients compared to controls [34,35], which is reflective of neurodegeneration and microvascular impairment in the retina [36]. Since both structures reflect neurodegeneration and microvascular dysfunction in dementia, the significant correlation between the FAZ area and MTA may suggest that FAZ changes in EOD may reflect atrophy in the medial temporal lobe.

We showed that DWMH scores assessed with the Fazekas scale significantly correlated with SVC and DVC densities and FAZ area. DWMH arises from ischemic changes disorders in the brain [37]; it is linked with neuropathological changes caused by disruptions in nerve fibers which result in ischemic changes [38]. OCTA reports on dementia patients showed reduced SVC and DVC densities and enlarged FAZ area, which are thought to be indicative of vessel wall dysfunction, neurodegeneration, and retinal microvascular hypoperfusion [14,15,16,39]. Similar processes that are linked with ischemic causes may lead to deep white matter lesions [40,41] suggesting similar mechanisms on a microvascular level in both the retina and brain of EOD patients. On the other hand, a previous report showed DWMH significantly correlated with cerebral Aß accumulation [42]. Similarly, in patients with dementia, Aß deposits were found in the ganglion cell layer (GCL), inner nuclear layer (INL) and outer nuclear layer (ONL) [43], which make up the SVC, DVC and FAZ. This suggests that there may be a link between DWMH and SVC, DVC and FAZ in the retina of EOD patients. 

We also found that SVC density in EOD patients significantly correlated with PWMH scores assessed with the Fazekas scale. Retinal imaging studies using fundus photography showed that changes in the retinal vein (located in the SVC) are associated with cardiovascular risk factors such as hypertension [44,45] and diabetes [46,47], which are linked with dementia. Besides, it has been hypothesized that enlargement of the retinal venules reflects endothelial dysfunction [48], which is a classical indicator of ischemia in the retina and hypoperfusion. Since the SVC reflects the microvasculature seen in fundus photography, our reports are in line with the hypothesis that microvascular impairment in the brain could be reflected in the SVC. On the other hand, PWMH is suggested to be vulnerable to decreases in blood flow due to it being situated in the arterial region of the brain. Similarly, the SVC, which is located in the superior region of the retina, is responsible for arterial circulation in the retina [49]. Previous OCTA reports have shown reduced SVC density (arterial circulation) in dementia patients compared with controls, suggesting that SVC changes may reflect dysfunction in the arterial circulation of the retina, which may ultimately reflect PWMH in EOD patients.

## 5. Limitation and Strengths

Our current study had several limitations. Our enrolled EOD patients did not undergo a comprehensive ophthalmological examination, thus subtle ophthalmological disorders such as diabetic retinopathy, mild cataracts, and high intraocular pressure cannot be ruled out completely. OCTA angiograms of all patients were evaluated by an ophthalmologist specialist to establish any relevant disorder. Another limitation is the lack of volumetric MRI data to further support clinical MRI ratings and the lack of supportive biomarkers of dementia such as amyloid PET or cerebrospinal fluid amyloid-tau measurements that may have further served to reinforce clinical diagnosis. The observational cross-sectional study design is another limitation of our study. Our current study focused on retinal microvasculature and its association with radiological markers in EOD. This is a strength of our study as the retinal microvasculature reflects the cerebral microcirculation, thus suggesting that microvascular impairment is associated with radiological markers in EOD. 

## 6. Conclusions

In conclusion, our report showed FAZ area in EOD was significantly correlated with MTA scores, while retinal microvasculature was correlated with white matter hyperintensities measured by the Fazekas scale. This suggests that microvascular changes in the retina reflect microstructural changes in the brain. Taken together, our report suggests that OCTA imaging might provide useful information on the role of microvasculature in radiological markers of EOD and enable the evaluation of purported therapy. Since our study focused on in vivo quantitative measurements of the retinal microvasculature, histological comparisons would be worthwhile to assess these microvascular changes and the correlation neuroimaging parameters. 

## Figures and Tables

**Table 1:** Baseline characteristics of EOD patients.

	Descriptive
Number	63
Gender, M	27
Age, years	60.43 ± 5.80
Systolic blood pressure, mmHg	126.84 ± 11.09
Diastolic blood pressure, mmHg	78.39 ± 7.21
Hypertension, n	11
Diabetes, n	4
Education, years	9 (6 -12)
Duration, years	2 (1–3)
MMSE	14 (9–20)
MoCA	9 (6–15)
FAZ area, mm²	0.35 ± 0.13
SVC, %	42.37 ± 5.52
DVC, %	48.47 ± 4.50
MTA	2 (1–2)
PCA	1 (1–2)
GCA	1 (1–2)
PWMH	1 (0–1)
DWMH	1 (0–1)
CAA, n	5

MMSE: Mini-Mental State Examination; MoCA: Montreal Cognitive Assessment; FAZ: foveal avascular zone; SVC: superficial vascular complex; DVC: deep vascular complex; MTA: medial temporal lobe atrophy; PCA: parietal cortical atrophy; GCA: global cortical atrophy; PWMH: periventricular white matter hyperintensities; DWMH: deep white matter hyperintensities; CAA: cerebral amyloid angiopathy.

**Table 2:** Correlation between SS-OCTA parameters and radiological indicators of dementia.

	SVC			DVC			FAZ		
	B	SE	*p*-Value	B	SE	*p*-Value	B	SE	*p*-Value
MTA	−0.024	0.027	0.374	−0.046	0.037	0.208	2.362	1.095	0.031
PCA	0.001	0.024	0.953	0.025	0.03	0.398	−0.637	0.924	0.491
GCA	−0.021	0.02	0.285	−0.013	0.023	0.562	0.894	0.584	0.126
PWMH	0.027	0.013	0.032	0.032	0.017	0.052	−0.5	0.686	0.466
DWMH	0.037	0.014	0.007	0.038	0.016	0.018	−1.715	0.535	0.001

Data were adjusted for age, gender, hypertension, diabetes, and CAA. MTA: medial temporal lobe atrophy; PCA: parietal cortical atrophy; GCA: global cortical atrophy; PWMH: periventricular white matter hyperintensities; DWMH: deep white matter hyperintensities; CAA: cerebral amyloid angiopathy; SVC: superficial vascular complex; DVC: deep vascular complex; FAZ: foveal avascular zone; B: beta coefficient; SE: standard error.

## Data Availability

The data that support the finding of this study are available on request from the corresponding author.
